# A rare clinical entity as large intrarenal abscess in a typeII diabetic patient due to *Candida* *tropicalis*: Case report

**DOI:** 10.18502/cmm.5.4.2150

**Published:** 2019

**Authors:** Abhilash Chandra, Namrata Rao, Anupam Das, Manodeep Sen

**Affiliations:** 1 Department of Nephrology, Dr. Ram Manohar Lohia Institute of Medical Sciences, Vibhuti Khand, Gomti Nagar, Lucknow, India; 2 Department of Microbiology, Dr. Ram Manohar Lohia Institute of Medical Sciences, Vibhuti Khand, Gomti Nagar, Lucknow, India

**Keywords:** Abscess, Candida tropicalis, Diabetes, Fungal, Renal

## Abstract

**Background and Purpose:**

Fungal renal abscesses are rare entities associated with significant morbidity and mortality. Affected kidneys can have microabscess, pyelonephritis, pyonephrosis, or papillary necrosis.

**Case report:**

Herein, we reported an unusual case of a large renal abscess cause by *Candida tropicalis* in a diabetic patient. The entity presented as a lump in the abdomen and later was diagnosed to be an abscess on computed tomography scan. *Candida tropicalis* was confirmed on the culture of the aspirate. The abscess was successfully treated by percutaneous drainage and administration of amphotericin B deoxycholate.

**Conclusion:**

*Candida tropicalis* is now a global concern because of its rising prevalence and high virulence. The growing resistance of this *Candida* species to azoles, as in our case, calls for a more judicious usage of antifungal agents. Empirical therapy with either amphotericin or echinocandins is an option in case of high azole resistance. This case highlights the importance of timely diagnosis and implementation of aggressive management in cases suffering from fungal abscesses.

## Introduction

Fungal renal abscesses are rare entities occurring either through hematogenous spread or ascending infection [1]. These entities are associated with significant morbidity and mortality [2]. Variability of the clinical presentations of such abscesses, including non-specific signs and symptoms, may delay their diagnosis.

Immunocompromised status predisposes the individuals to such infections, therefore calling for a high index of suspicion with stress on early diagnosis and management. *Candida tropicalis,* as a fast emerging crucial *Candida* species worldwide, is known to cause invasive life-threatening systemic infections [3, 4]. 

Herein, we present a rare case of a large renal abscess caused by *C. tropicalis* in the right kidney in a type II diabetic patient with renal dysfunction, which was effectively managed with antifungal admiration and percutaneous drainage. In addition to this species, *Mucormycosis* and *Aspergillosis* are the other common agents for the development of fungal infections involving the kidney. There are also few reports introducing *Cryptococcosis* and *Histoplasmosis* as other pathogenic agents [5].

## Case report

A type II diabetic 45-year-old male with the complaints of right flank pain and swelling with a high-grade fever for the last 15 days was admitted to the department of nephrology of a tertiary care hospital in Northern India, in March 2019. Occasionally, he also had burning sensation during micturition. On physical examination, the patient was febrile (102°F/38.9°C) with a heart rate of 101/min and blood pressure of 90/60 mmHg. There was a lump in the right side of the abdomen, which was slightly tender, extending up to 7 cm below the right subcostal margin. The margins were ill-defined. The lump was soft and ballotable with no pulsations. 

**Figure 1:**
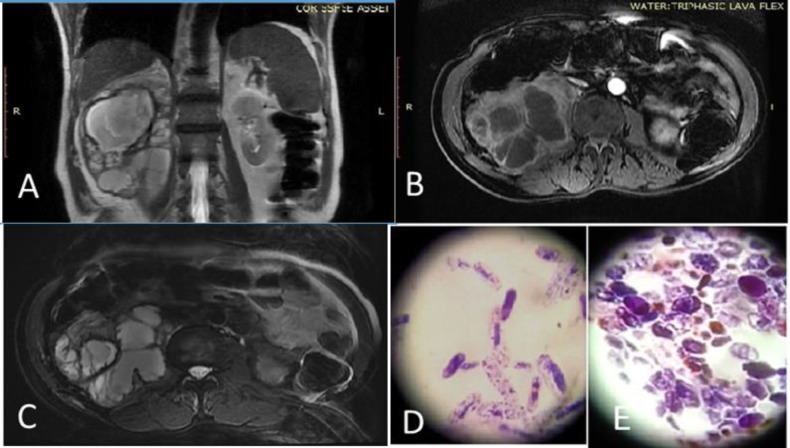
A) T2 coronal showing positive hyperintense fluid within the renal fossa with maintained cortical renal capsule, B) axial fat supressed T1 (Post contrast images showing the moderate contrast enhancement of the wall, septa, and loculation within the abscess) C) axial heavily weighted T2 images showing hyperintense thick fluid collection with multiple irregular hypointense septations, D) elongated budding yeast cells of *Candida tropicalis* in gram stain (100X magnification), E) budding yeast cells in gram stain (100X magnification)

Blood test reports revealed low hemoglobin level (9 gm/dL), polymorphonuclear leucocytosis of 16500/mm^3^, and high serum creatinine (4.3 mg/dL). In addition, the HbA1c level was 8%. Urine test indicated 100 mg/dl proteinuria, 30-40 white blood cells per high power field (hpf), and 7-8 red blood cells per hpf. Furthermore, the urine culture and blood culture (VesaTREK automated blood culture system, USA) were sterile. With renal dysfunction and a suspicion of renal mass lesion, the patient was subjected to a non-contrast computed tomography (CT) scan, which revealed a large-size intrarenal abscess (Figure 1 A, B, C). An ultrasound-guided percutaneous drainage was performed which yielded around 3 L of thick brownish material. Septa were torn during catheter insertion, and extra number of holes were created in the catheter to drain pus from different compartments. Aspirate culture grew *C. tropicalis* in Saboroud dextrose media (High Media, India; Figure 1 D, E) which was identified by the Vitek 2 Compact automated system (Biomerieux, Durham USA). 

The susceptibility of the isolate to echinocandins (i.e., caspofungin and micafungin), amphotericin B, and fluconazole was tested using the Vitek 2 Compact. The results revealed the sensitivity of the isolate to caspofungin (MIC<0.12 µg/ml), micafungin (MIC<0.06 µg/ml), and amphotericin (MIC<0.25 µg /ml) and its resistance to fluconazole (MIC=8 µg/ml). Therefore, the patient was administered with amphotericin B deoxycholate (1 mg/kg/day) for 3 weeks. He showed improvement within a week of initiating antifungal therapy in terms of settling of fever and serum creatinine showing a downward trend. 

The patient was discharged with an advice to attend the outpatient clinic for follow-up on a weekly basis for 2 weeks, followed by bi-weekly referral for 4 weeks and another referral 3 months after hospital discharge. He was completely asymptomatic without any evidence regarding the recurrence of fungal infection with a serum creatinine of 1.8 mg/dl at the end of 3 months. 

## Discussion

Predisposing factors for *Candida* infections include prolonged urinary catheterisation, presence of central venous catheter, use of immunosuppressive medications, diabetes mellitus as comorbidity, malignancy, indiscriminate use of broad-spectrum antibiotics (altering the natural flora of microorganisms), and abnormalities of the urinary tract (e.g., neurogenic bladder and obstructive lesions) [2]. 

Our patient had longstanding and poorly controlled diabetes mellitus which was the risk factor in this case. Diabetes provides a favourable condition for infective organisms via neutrophil dysfunction and increased adherence of the bacteria to uroepithelial cells [6]. In the current era of advanced diagnostic modalities, interventions, and antimicrobials, affliction with diabetes dose not influence the overall mortality in renal abscess cases [7, 8]. However, it can elongate the patient’s hospital stay [8].

*Candida tropicalis* is now a global concern because of its rising prevalence and high virulence. This strain has certain special characteristics which make it stand out among other *Candida *species. In this regard, this species produces biofilm to a much larger extent than other *Candida *species [9]. It is also armed with higher proteinase and hemolytic activity [10]. The virulence of this *Candida *species depends upon the specific strain and host defence [11]. 

The other concern about *C. tropicalis* is its growing resistance to azoles. The results of a study conducted in Asia Pacific region by Tan et al. demonstrated a low fluconazole susceptibility (74%) for *C. tropicalis* [12]. A fluconazole resistance pattern was also found in our case. Overexpression of *CDR1*, *MDR1*, and *ERG11* genes in the clinical isolates, along with the previous history of azole usage, has been proposed as the mechanism behind this phenomenon [13]. This issue calls for a more judicious usage of azoles, along with continuous surveillance for the epidemiology and susceptibility pattern of these fungal organisms [14]. 

Candidemia is a more frequent reported form of systemic infection. Similar to our case experiencing single organ involvement, there are reports of individually affected organs manifesting as endocarditis, septic arthritis and endophthalmitis, gastrointestinal tract infection, and neurocandidiasis. There are also reports regarding the histopathological and radiological evidence of low-grade infection involving the liver, spleen, and kidney [15, 16]. In terms of invasive infections, *C. tropicalis* has been associated with higher mortality as compared to other *Candida* non-*albicans* [4]. 

There are pathologic studies in the literature addressing abscess formation, emphysematous pyelonephritis, papillary necrosis, pyonephrosis, and vascular involvement leading to infarction or necrosis and development of fungal ball in the kidneys [5, 17]. In this regard, Zhao Song et al. reported on the incidence of candidiasis in the kidney presenting as a mass lesion [18]. However, to the best of our knowledge, there is no study reporting an abscess of this size.

Clinically, this condition may present with fever, dysuria, flank pain or frank hematuria, and renal failure [5]. If the infection occurs by an ascending route, the presentations are more likely to be sloughing and debris or fungal ball formation in the pelvicalyceal system. However, the hematogenous route is more likely to lead to the formation of abscess or micro abscesses in the kidney with the involvement of other organs. 

Our case was a systemic infection with a possible hematogenous route leading to a large intrarenal abscess formation. Imaging can render peculiar findings; in this regard, ultrasonic images can show the increase in the size of the kidney, echogenic debris in the pelvicalyceal system with thickening of the walls, caliectasis, loss of corticomedullary differentiation, papillitis, hydronephrosis, or space occupying lesion. The CT scan results can be confirmative of ultrasonic findings showing the additional features of perinephric fat stranding, thickening of Gerota’s fascia, abscess formation, or papillary necrosis. Diagnosis of this condition is primarily based on histopathological examinations, including microscopic examination, culture, or other uncommonly performed antigen-antibody testing/molecular methods [19]

Effective management of such abscesses requires the implementation of combined medical and surgical interventions. Siegel has advocated surgical management for the abscesses measuring above 3 cm [20]. Bamberger reported a worse outcome in patients with the abscess size of more than 5 cm managed with medical treatment alone [21]. Empirical therapy with either amphotericin or echinocandins, along with USG/CT-guided drainage, is the best approach for such cases as demonstrated in our case. Furthermore, antifungal treatment can later be modified as per the sensitivity pattern.

## Conclusion

Considering the rarity of primary renal candidiasis and its wide spectrum of presentation, this case adds another chapter to the existing data. Successful management involves clinical suspicion, implementation of imaging, and microbiological analysis, followed by aggressive surgical and/or medical treatment. An equally important issue is the identification of individual *Candida *species given their variable sensitivity to azoles. 

## Author’s contribution

A.C. contributed to the study concept, wrote the first draft of the manuscript, and managed the project. N.R., A.D., and M.S. performed the critical revision of the manuscript and provided the practical support.

## Conflicts of interest

None of the authors have any conflicts of interest to declare.

## Financial disclosure

The authors declare no conflicts of interest. The authors are responsible for the content and writing of the paper.
